# Modulation of gut microbiota: The effects of a fruits and vegetables supplement

**DOI:** 10.3389/fnut.2022.930883

**Published:** 2022-09-23

**Authors:** Arun Prasath Lakshmanan, Alessandra Mingione, Francesca Pivari, Elena Dogliotti, Caterina Brasacchio, Selvasankar Murugesan, Daniele Cusi, Monica Lazzaroni, Laura Soldati, Annalisa Terranegra

**Affiliations:** ^1^Precision Nutrition, Research Department, Sidra Medicine, Doha, Qatar; ^2^Department of Biomedical and Clinical Sciences L. Sacco, University of Milan, Milan, Italy; ^3^Department of Health Sciences, University of Milan, Milan, Italy; ^4^Fondazione Umberto Veronesi, Milan, Italy; ^5^Microbiome and Host-Microbes Interactions Lab, Research Department, Sidra Medicine, Doha, Qatar; ^6^Institute of Biomedical Technologies, Italian National Research Council, Milan, Italy; ^7^Bio4Dreams Scientific Unit, Bio4Dreams-Business Nursery for Life Sciences, Bio4Dreams S.p.A., Milan, Italy; ^8^Laboratory of Clinical Pathology, Foundation IRCCS Neurological Institute C. Besta, Milan, Italy

**Keywords:** diet supplements, gut microbiota, antioxidant capacity, fruits and vegetables, SCFA

## Abstract

The consumption of an optimal amount of fruits and vegetables is known to improve physical fitness and physiological body functions. Healthy eating habits, including intake of fruits and vegetables, can modify gut microbiota. This study aimed to demonstrate the effectiveness of a formulated fruit and vegetable supplement (FVS) in modulating the antioxidant capacity and the gut microbiota composition. We enrolled 30 healthy volunteer subjects, matched for age, gender, BMI, and smoking habits, and randomized them into the FVS and the placebo (PLA) groups. Among the serum vitamins, the folic acid level was significantly higher (*p* = 0.001) in the FVS group than in the PLA group, whereas the vitamin B2 level was significantly higher in the PLA group than in the FVS group (*p* = 0.028). The antioxidant capacity, measured by using the oxygen radical absorbance capacity (ORAC) method, was also slightly higher in the FVS group than in the PLA group but did not reach statistical significance. The dietary intake, assessed by 24-h recalls, did not show any significant changes after the supplementation in both the groups. The gut microbiome composition, measured by 16S rDNA sequencing, showed no difference in both alpha and beta diversities, whereas the LEfse analysis revealed a microbial shift after the treatment, with a decreased abundance of the genus *Ruminococcus* from the Lachnospiraceae family (*p* = 0.009), and the unclassified genus from the family Erysipelotrichaceae (UC36, *p* = 0.003) in the FVS group compared with the PLA group (confirmed by SIAMCAT analysis, AUC = 74.1%). With a minor effect, the genus *Faecalibacterium* and unclassified genus and family from the order Lactobacillales (UC31) were also increased in the FVS group compared with the PLA group (*p* = 0.0474, *p* = 0.0352, respectively). SCFA measurement by gas chromatography–mass spectrometry showed an increased level of 2-methylbutyrate in the FVS group compared with the PLA group (*p* = 0.0385). Finally, the Spearman correlation analysis showed that in the FVS group, the genus *Faecalibacterium* positively correlated with 2-methyl butyrate (*p* = 0.040). In the PLA group, none of the significant bacteria correlated with either SCFA or serum biomarkers. The network analysis confirmed the positive correlation between genus *Faecalibacterium* and 2-methyl butyrate. We can conclude that the FVS in healthy individuals modified the gut microbiota composition and metabolites, and it can potentially contribute to reduce the pro-inflammatory response along with the antioxidant capacity.

## Introduction

The reports from clinical and preclinical studies suggest that consumption of an optimal and routine intake of fruits and vegetables, which are rich in vitamins, minerals, fibers, and active molecules, is beneficial for the overall physical fitness, immune functions, and normal physiological functions of the body (1–4). Fruits and vegetables contain a high level of antioxidants, which counteract the action of free radicals. Unfortunately, the continuous exposure to free radicals, present in many harmful substances, can cause cell damage, favoring the onset of various diseases. A positive correlation between the continuous intake of fruits and vegetables and the reduced risk of vascular diseases and cancers has been established (5–7). Fruit and vegetable nutrients, such as fiber and polyphenols, are known to affect the gut microbial composition (4, 8). Gut microbiome realizes mutualistic relations with the gut, influencing the maturation of the immune system, modulating the responses to epithelial cell injury, affecting energy balance, and protecting against physiologic stress (9). According to the reports from world-renowned national and international monitoring agencies that deal with diet and nutrition, consumption of an adequate amount of fruits and vegetables could change the world map of major chronic diseases (cardiovascular disease, stroke, cancer, osteoporosis, diabetes, metabolic diseases, etc.). Nowadays, the majority of the world population is exposed to Western diets, characterized by an over-intake of saturated and omega-6 fatty acids and reduced intake of omega-3 fatty acids, fruits, vegetables, and fibers (10). A Western-like dietary pattern, together with a sedentary lifestyle, leads to several inflammatory-related disorders, such as metabolic syndrome, cardiovascular disease, and neurodegenerative diseases (11). Most of these disorders are also associated with alterations in microbiota composition in humans, especially those with reduced bacterial richness and diversity (12). In this context, the Mediterranean diet (MD) is recognized as one of the healthiest diets worldwide as it contains a high proportion of fiber, antioxidants, and polyphenols, present in vegetables, fruits, pulses, and extra-virgin olive oil, which are strongly associated with a reduced risk of developing non-communicable diseases related to Western diet and lifestyle (13).

It has been estimated that the consumption of 500–800 g/day of fruits and vegetables would avoid over 5.6 and 7.8 million premature deaths annually, a 30% reduction in the relative risk for coronary heart disease, a 28% reduction in stroke and cardiovascular diseases, and a 14% reduction in total cancer risk with 500–600 g/day of fruits and vegetables (14–16). Although antioxidant intake through whole foods, as well as low- to moderate-dose nutritional supplements, is generally considered to provide health-enhancing benefits, higher dose antioxidant supplements intake is somewhat controversial (17). A recent interventional study conducted by Ren et al. used a well-balanced combination of antioxidant nutrients, which provide an increased antioxidant defense (18). Thus, the proposed study aimed to demonstrate the effectiveness of formulated fruit and vegetable supplementation in modulating the gut microbiome composition and explore the potential mechanism that involves the gut microbiome compositional change in healthy subjects.

## Materials and methods

### Human subjects and the study protocol

Healthy adult volunteers (both male and female, *N* = 30) aged 18–65 years were recruited during the first phase of the study at San Paolo Hospital, Milan, Italy. The study is randomized, double-blinded, and placebo-controlled, and the volunteers were randomly assigned to the treatment or placebo group, with 15 healthy subjects in each group. The randomization was conducted through open-source software.^1^ The flow diagram of the present study is given in the Supplementary material (Supplementary Figure 1). The subjects who were given the fruit and vegetable supplement are labeled the FVS group, and the subjects who were given placebo are labeled the PLA group. The study duration was 6 weeks, the lifestyle questionnaire was used, and the measurement of BMI and waist circumference was taken before and after the supplement/placebo treatment. The assessment of food intake was carried out by using the 24-h food recall method before and after the treatment. The blood and stool samples were collected from each participant before and after the supplement/placebo treatment. The study was approved by the Ethical Committee of Interaziendale Milan Area A, with the approval number 156/ST/2014.

### Diet supplements

The diet supplement contains a mix of fruits and vegetables, packed in stick packs to provide an equal amount of supplements to each subject, distributed by L’Angelica Istituto Erboristico, and named fruit and vegetable supplement (FVS). The placebo supplement was also packed in stick packs without the content of fruits and vegetables and named PLA (Supplementary Table 1). Each subject of both FVS and PLA groups was given two stick packs per day for a total of 6 weeks. All the subjects were recruited in the same season to avoid the effect of different seasonal foods.

### Oxygen radical absorbance capacity assay

The antioxidant capacity of the plasma samples was assessed by using an oxygen radical absorbance capacity (ORAC) Antioxidant Assay Kit (Zen Bio). Antioxidants present in the samples can inhibit the peroxyl radical-mediated oxidation of fluorescein formed by the breakdown of 2,2’-azobis-2-methyl-propanimidamide dihydrochloride (AAPH) at 37°C. The fluorescence signal is measured during 60 min by determining the Ex480 nm/Em520 nm ratio. The concentration of antioxidants in the plasma sample is proportional to the fluorescence intensity and is assessed by comparing the net area under the curve to that of a known antioxidant, Trolox. The ORAC of plasma samples, before and after FVS intake, was expressed as micromolar Trolox equivalents (μMTE).

### Measurement of serum levels of vitamins A, E, B2, B6, and K, and folate

Blood samples were collected before and after the start of FVS treatment. The measurement of serum levels of vitamins A, E B2, and B6 was carried out using a high-performance liquid chromatography (HPLC) system using commercial reagent kits (Chromsystems Instruments & Chemicals GmbH, Munich, Germany) according to the manufacturer’s guidelines. Vitamin K was determined using the ion-selective electrode. Serum folate was measured using the standard electrochemiluminescence assay (Roche Diagnostics, Basel, Switzerland).

### Dietary data analysis

The expert nutritionist collected the dietary intake of each participant, at baseline and after 6 weeks of the FVS, during a structured interview using a 24-h dietary recall to estimate subjects’ food consumption and the Scotti Bassani Photographic Atlas to better estimate food portions.^2^ The Diet Monitoring Solution (DMS) web platform was used to collect participants’ nutritional data, dietary micronutrients, and macronutrients (19).

### Bacterial DNA extraction from stool samples

Stool samples were self-collected by each participant in Norgen Stool Nucleic Acid Collection and Transport Tube (Norgen Biotek Corp., Toronto, Canada) and were stored at a –80^°^C freezer until further use. Bacterial DNA extraction was performed using QIAamp^®^ Fast DNA Stool Mini Kit (Qiagen, Germany) according to the manufacturer’s instructions. The quantity and quality of the extracted DNA were checked by using NanoDrop One (Thermo Fisher Scientific,Waltham, MA, United States).

### 16S rDNA sequencing

The 16S rDNA library preparation and sequencing were performed according to the manufacturer’s instructions (MiSeq system, Illumina, San Diego, CA, United States), as described previously (20). In brief, the extracted genomic DNA was amplified using the primers that target the v3-v4 regions of the 16S rDNA gene. The amplified product was then cleaned up using AMPure XP magnetic beads (Beckman Coulter, Brea, CA, United States), and the product was indexed using the Nextera XT primer (Illumina, San Diego, CA, United States). Again, the product was cleaned up using magnetic beads. The size and quantity of the prepared library were estimated using an Agilent High Sensitivity Kit and a Qubit dsDNA HS Assay Kit, respectively. The pooled library and Phix control were denatured using 0.2 N NaOH as per the manufacturer’s protocol. Finally, the sample was sequenced using the MiSeq Reagent v3 (600 cycles) Kit (Illumina, San Diego, CA, United States) according to the manufacturer’s instructions. Base calling was directly carried out on MiSeq. The raw data were demultiplexed using MiSeq Reporter on Illumina MiSeq. The PEAR tool was used to merge both forward and reverse end sequences for each sample (21), and the reads with a high-quality score of 30 and above were selected using the Trimmomatic tool (22). FASTQ files were converted into FASTA files using QIIME v1.9.0 (Quantitative Insights Into Microbial Ecology) pipeline (23). Operational taxonomic units (OTUs) were obtained by aligning the sequence against the Greengenes database (gg_13_08) with a confidence threshold of 97% (24).

### Short-chain fatty acid analysis by liquid chromatography–tandem mass spectrometry

The analysis of short-chain fatty acid (SCFA) was performed by adaptation of the method published by Han et al. (25). In brief, the collected stool sample was homogenized using a spatula, weighed, and diluted with 50% aqueous acetonitrile (Fluka, Thermo Fisher Scientific, Waltham, MA, United States). A portion of the supernatant was taken for further analysis, along with mixed standard calibration solutions representing a range of concentrations for each fatty acid. All SCFAs from C2 to C6 along with any iso- and anteiso-methyl branched chain fatty acids were tested. The samples and standards were derivatized with 3-nitrophenylhydrazine (Sigma Aldrich, St. Louis, MO, United States) and then diluted by a factor of 10 with 10% aqueous acetonitrile. An internal standard (a mixture of SCFA derivatized as above with ^13^C_6_-3-nitrophenylhydrazine (IsoSciences, PA, United States) was added. To test if the stool matrix influenced the recovery, controls were prepared by spiking isotopically labeled straight-chain SCFA derivatized with ^13^C_6_-3-nitrophenylhydrazine to a mixture of stool sample supernatants from this study. These were analyzed along with the same mixture of isotopically labeled SCFA prepared in 50% aqueous acetonitrile, and a comparison was made. All samples were analyzed using a liquid chromatography–triple quadrupole mass spectrometer operated in negative ion scheduled MRM mode. A C18 column allowed the chromatographic separation of all derivatized SCFAs. The peak area for all chromatographic peaks was calculated and used for generating calibration curves and for calculating unknown concentrations of SCFA in stool.

### Computational analysis of gut microbiome

#### Taxa summary

We used a paired-end read merger (PEAR, v0.9.8) tool to merge both forward and reverse end sequences for each sample (21), and the reads with a high-quality score of 30 and above (≥30) were selected using the Trimmomatic (v0.36) tool (22). FASTQ files were converted into FASTA files using Quantitative Insights Into Microbial Ecology (QIIME, v1.9.0) pipeline (23). Operational taxonomic units (OTUs) were obtained by aligning the sequence against the Greengenes database (gg_13_08) with a confidence threshold of 97% (24).

#### Diversity indices

Alpha diversity refers to the estimation of both richness and abundance of species in a habitat or specific area or sample. Species richness refers to the number of species present in a sample, and species abundance means the number of individuals per species. Alpha diversity was estimated using observed (species richness) and Chao1 (rare species richness), and Shannon and Simpson methods (species abundance) by using the R package (Phyloseq and ggplot2), as described previously (26). Beta diversity refers to the measurement of difference in the microbial composition between two or more groups of samples, and it was presented as a principal coordinate analysis as proposed in QIIME v1.9.0, as described previously (26).

#### Identification of gut microbial markers

Linear discriminant analysis effect size (LEfSe) was used to obtain the gut microbial markers for each group, and it uses the non-parametric factorial Kruskal–Wallis (KW) sum-rank test to identify features with significant differential abundance with regard to different groups, followed by linear discriminant analysis (LDA) to calculate the effect size of each differentially abundant microbial features. Features are significant if the LDA value is > 2.0 and the *p*-value is < 0.01 (27). Differentially abundant microbial taxa were identified also using the analysis of composition of microbiomes (ANCOM), as previously described (28).

#### Association analysis of microbial markers and host phenotypes

We also performed an association analysis between microbial markers and host phenotypes using a Statistical Inference of Associations between Microbial Communities and host phenoTypes (SIAMCAT) machine learning tool, followed by “lasso” regression analysis according to the previously reported method by Wirbel et al. (29).

#### Prediction of functional pathway

Phylogenetic Investigation of Communities by Reconstruction of Unobserved States (PICRUSt) analysis is a bioinformatics software package designed to predict the metagenome functional content from marker gene surveys and full genomes, and it was performed according to the literature review of Langille et al. (30).

#### Network analysis

We performed microbial, diet, and SCFA correlation network analysis using a MetagenoNets webtool, as described previously by Nagpal et al. (31).

### Statistical analysis

Comparisons between the groups were performed by parametric (unpaired *t*-test) or non-parametric analyses (Mann–Whitney test) according to data distribution. Correlation analysis of microbial data with diet data and antioxidant blood parameters was performed using Spearman’s correlation method. All analyses were run on Prism Software version 9 (GraphPad, CA, United States). A value of *p* < 0.05 was considered statistically significant.

## Results

### Supplementation effect on serum vitamins, antioxidant capacity, and dietary intake levels

**FIGURE 1:**
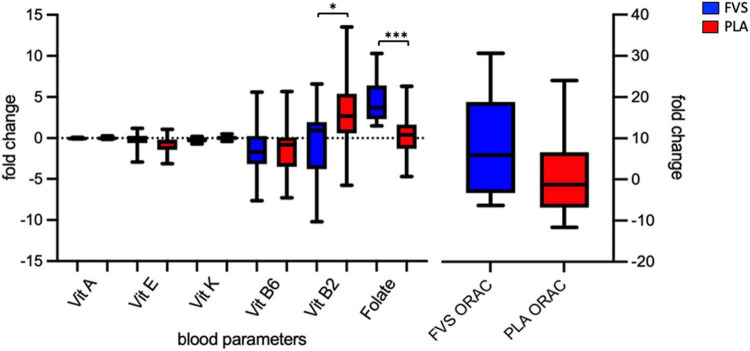
Serum biomarkers of the antioxidant capacity. Serum levels of vitamins A, E, K, B6, and B2 and folate, and antioxidant capacity in the FVS group (blue bars) and PLA group (red bars). The median and interquartile (IQR) ranges are showed in the box plots. FVS, *N* = 15, PLA, *N* = 14. *p < 0.05 and ^***^p < 0.001 when compared with the PLA group using the unpaired *t*-test.

We enrolled 30 healthy volunteers and randomized them into two groups (15 in the FVS group and 14 in the PLA group, with the dropout of 1 subject). The two groups did not differ in gender, age, BMI, and the smoking habits (Table 1). We measured the serum levels of vitamins A, B2, B6, E, and K, and folic acid in blood of the PLA and FVS groups, and we computed the changes in the levels after the supplementation for each subject. The level of folic acid was significantly higher in the FVS group than in the PLA group (unpaired *t*-test, *p* = 0.001). Interestingly, the level of serum vitamin B2 was significantly higher in the PLA group than in the FVS group (unpaired *t*-test, *p* = 0.028). There was no significant difference in the level of other vitamins. The level of ORAC was slightly higher in the FVS group but did not reach a statistical significance (Figure 1). We assessed the dietary intake of micronutrients and macronutrients in order to determine changes in the food habits, and the study participants in both the groups did not show any significant change in their dietary intake during the treatment (Supplementary Figure 2).

**TABLE 1:** Demographic data of the study participants.

	FVS	PLA
Females (n)	7	7
Males (n)	8	7
BMI (kg/m^2^)	22.60 ± 2.80	25.09 ± 5.04
Age (years)	42.79 ± 15.36	43.3 ± 14.49
Smokers	2	3

### Modifications in the gut microbial composition after supplementation

**FIGURE 2:**
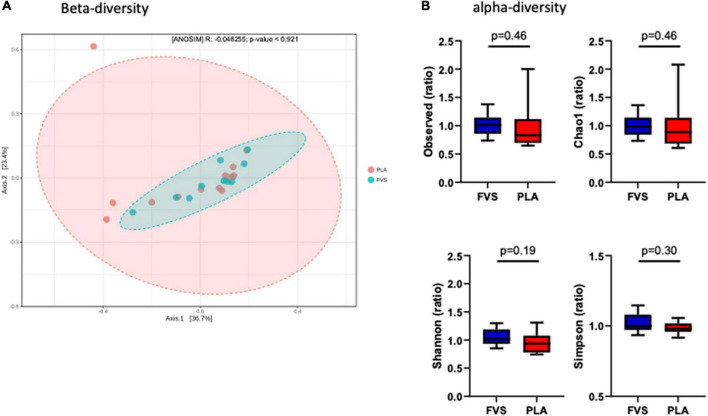
Gut microbial diversity in the FVS and PLA groups. **(A)** Beta diversity index was measured by using the Bray–Curtis method using principle coordinate analysis (PCoA) using the relative abundance of OTUs. The two variances explained by Axis.1 and Axis.2 are 36.7 and 23.4%, respectively. ANOSIM, analysis of similarity was not significant (*p* = 0.921). **(B)** Alpha diversity was measured by the four commonly used methods, such as observed, Chao1, Shannon, and Simpson methods. The box plots show interquartile (IQR) ranges with the median and whiskers. FVS, *N* = 15, PLA, *N* = 14. *p* < 0.05 considered statistically significant using Student’s *t*-test.

To determine the effect of the FVS on gut microbial composition, we performed 16S rDNA sequencing, and the results showed that there was no significant shift in the microbial diversity, which was evident from alpha and beta diversity estimation. Bray–Curtis-based principal coordinate analysis (PCoA) was performed to visually explore the similarity and variations between the microbial composition of the samples, and it showed that there was no visual separation between the PLA and FVS groups (Figure 2A). Alpha diversity analysis calculated using observed, Chao1, Shannon, and Simpson methods showed that there was no significant difference between the FVS and PLA groups after treatment (Figure 2B and Supplementary Figure 3).

**FIGURE 3:**
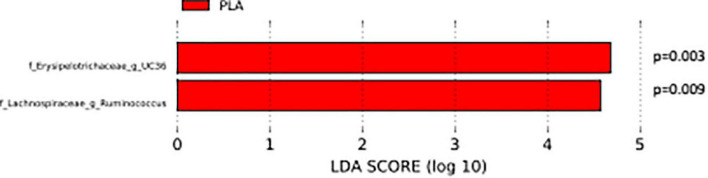
Gut microbial composition and microbial markers in the FVS and PLA groups. Gut microbial markers were measured by using the LEfSe analytical tool with a cutoff value of LDA > 2.0) in both the FVS and PLA groups. “g_UC” and “f_UC” represent unclassified bacteria at the genus level and family level, respectively.

**FIGURE 4:**
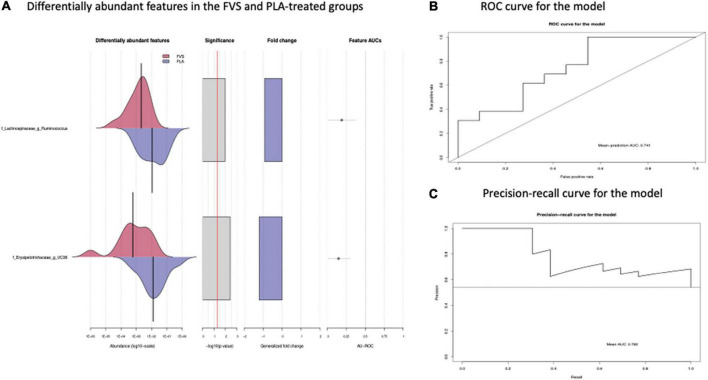
Validation of gut microbial markers using a machine learning tool—SIAMCAT analysis. **(A)** Gut microbial markers from LEfSe analysis were further validated using the SIAMCAT tool, which showed L-*Ruminococcus* and *Erysipelotrichaceae* family bacteria are significantly enriched in the PLA-treated group. **(B)** ROC curve for model, which displayed the cross-validation error as a receiver operating characteristic (ROC) curve with the 95% confidence interval. The area under the ROC (AUROC = 0.741) is given below the curve. The *x*-axis and *y*-axis represent false-positive and true-positive rates, respectively, for the tested markers, and **(C)** precision–recall curve for the model, which displays the mean AUC value of 0.782. An AUROC value of more than 0.7 is considered fairly good in terms of the discriminative ability of the test.

The analysis of the taxonomic composition revealed a shift of the microbial composition after the treatment. Applying the LefSe linear discriminant analysis, we found that the genus L-*Ruminococcus* (*p* = 0.009) and the unclassified genus from *Erysipelotrichaceae* family (*p* = 0.003) are enriched in the PLA group in comparison to the FVS group (Figure 3). The analysis of the composition of microbiomes (ANCOM) revealed that mostly L-*Ruminococcus* is the differential abundance bacteria between the FVS and PLA groups (Supplementary Figure 4). Also, the association analysis by using the SIAMCAT machine learning tool confirmed that L-*Ruminococcus* and unclassified bacteria from *Erysipelotrichaceae_UC36* family are significantly associated with the PLA group, with the robustness values of 97 and 98%, respectively. Furthermore, the mean overall prediction area under the curve (AUC) and mean AUC value are 74.1% and 78.2%, respectively (Figure 4). Comparison of the relative abundance at the phyla level and the top 30 genera from the two groups did not show statistical difference, except for the genus *Ruminococcus* and the *Erysipelotrichaceae_UC36.* Furthermore, the comparison at the genus level by using Mann–Whitney test showed a slightly significant increase in the relative abundance of the genus *Faecalibacterium* and unclassified genus and family from the order of *Lactobacillales* (UC31) in the FVS group compared with the PLA group (*p* = 0.0474 and *p* = 0.0352, respectively); whereas a significant increase in the relative abundance of genus L-*Ruminococcus*, *Lachnobacterium*, and unclassified genus from the family *Erysipelotrichaceae* (UC36) were found in the PLA group compared with the FVS group (*p* = 0.0003, *p* = 0.0010, and *p* = 0.0059, respectively) (Supplementary Figures 5, 6).

### Correlation network analysis of the gut microbial communities in the fruit and vegetable supplement and placebo groups

**FIGURE 5:**
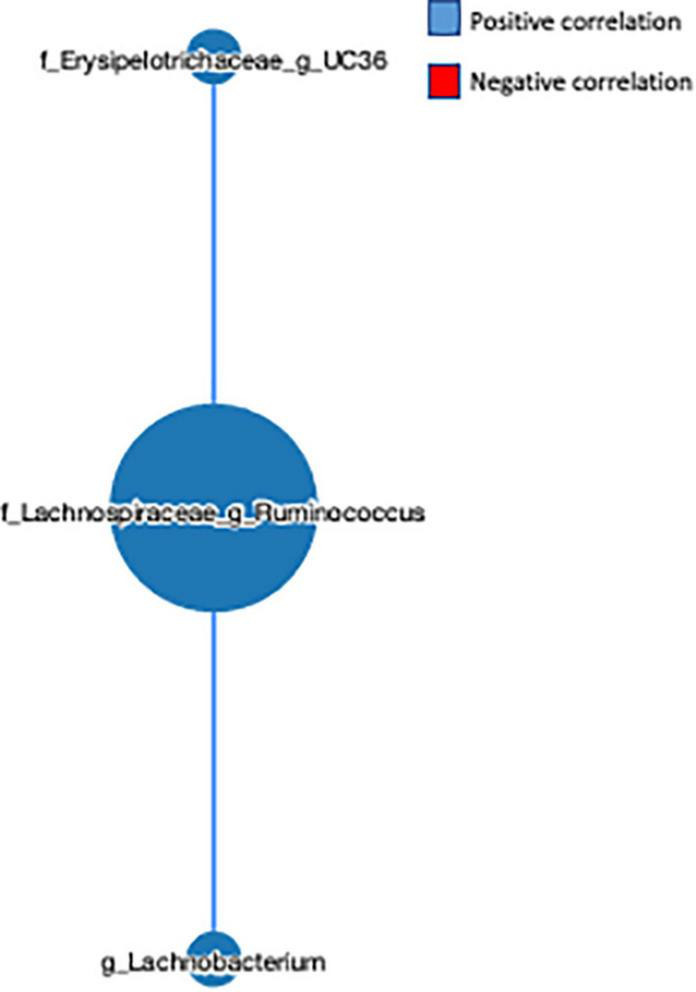
Microbial network analysis performed using a MetagenoNets webtool. The analysis revealed that L-*Ruminococcus* positively regulates the unclassified bacteria from the Erysipelotrichaceae family and the genus *Lachnobacterium*. Blue color indicates a positive correlation, whereas red color indicates a negative correlation.

The network-based approach is widely used in microbiome analysis as it provides greater information about the co-occurrence nature and interaction of the microbial communities (32). Here, we observed that the genus L-*Ruminococcus* positively correlated with the genus *Lachnobacterium* (*r* = 0.454) and unclassified genus from the Erysipelotrichaceae family (*r* = 0.553) (Figure 5), indicating that the genus L-*Ruminococcus* co-existed with the other two bacteria, and the FVS that modifies the abundance of L-*Ruminococcus* could positively affect the abundance of other two bacteria.

### Changes in the bacterial metabolic pathways and metabolites after supplementation

**FIGURE 6:**
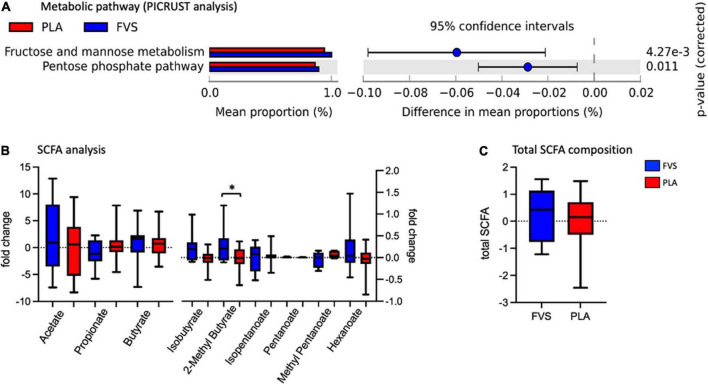
Predicted functional pathways and metabolite composition in the FVS and PLA groups. **(A)** Predicted functional pathways are obtained using the PICRUSt tool based on the bacterial abundance measured as mean proportion in the FVS and PLA groups. **(B,C)** SCFA composition was measured using liquid chromatography–tandem mass spectrometry. Single SCFAs are shown in panel b, and the total of the SCFA in panel c. The median and interquartile (IQR) ranges are showed in box plots. FVS, *N* = 15, PLA, *N* = 14. *p < 0.05 when compared with the PLA group using Student’s *t*-test.

The metabolic pathways predicted by PICRUSt revealed that the FVS group has a higher level of fructose and mannose metabolism and pentose phosphate pathways than the PLA group (Figure 6A). The measurement of various SCFAs clearly indicated that only the 2-methyl butyrate level was significantly (unpaired *t*-test, *p* = 0.0385) higher in the FVS group than in the PLA group (Figure 6B). The total amount of SCFA was higher in the FVS group but did not reach a statistical difference since the rest of the SCFAs, from acetate to hexanoate, were not significantly altered between the two groups (Figure 6C).

### Correlation analysis between the microbiome, SFCA, and serum biomarkers

**FIGURE 7:**
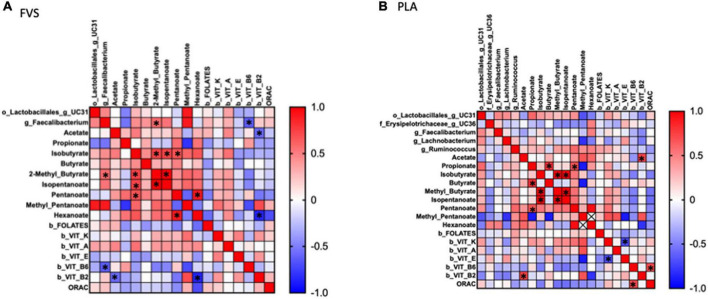
Correlation analysis between gut microbiome, SCFA, and serum biomarkers in the FVS and PLA groups. Spearman correlation was performed to analyze the interaction between significant microbial genus, SCFA, and serum biomarkers in both the FVS group **(A)** and PLA group **(B)**. Red to blue color scale indicates a positive to a negative correlation, respectively. FVS, *N* = 15, PLA, *N* = 14. *p < 0.05 when compared with the PLA group.

**FIGURE 8:**
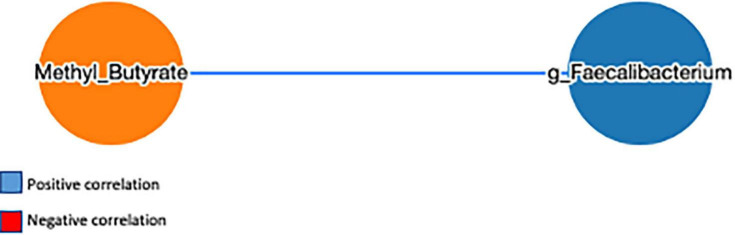
Network analysis performed using a MetagenoNets webtool. Analysis revealed that only 2-methyl butyrate positively correlated with the genus *Faecalibacterium* among all significant bacteria, diet, and SCFA. Blue color indicates a positive correlation, whereas red color indicates a negative correlation.

To explore the effect of microbiota on SCFA and serum biomarkers, we performed a Spearman correlation analysis including the significant bacteria for each group, all the SCFAs, and the serum levels of vitamins and ORAC. In the FVS group, the genus *Faecalibacterium* positively correlated with 2-methyl butyrate (*p* = 0.040) and negatively correlated with the serum level of vitamin B6 (*p* = 0.022). 2-Methyl butyrate positively correlated with isobutyrate and isopentanoate (*p* = 0.006 and *p* = 4.175e^–009^, respectively), and pentanoate with hexanoate (*p* = 0.005). Finally, vitamin B2 negatively correlated with acetate and hexanoate (*p* = 0.038, *p* = 0.011, respectively) (Figure 7A). In the PLA group, none of the significant bacteria correlated with either SCFA or serum biomarkers. Among the SCFAs, propionate positively correlated with butyrate and pentanoate (*p* = 0.003 and *p* = 0.027, respectively), isobutyrate with 2-methyl butyrate and isopentanoate (*p* = 5.858e^–007^ and *p* = 6.430e^–007^, respectively), isopentanoate with the 2-methyl butyrate (*p* = 8.351e^–009^). Finally, vitamin K negatively correlated with vitamin E (*p* = 0.007), and vitamin B6 positively correlated with ORAC (*p* = 0.006). Intriguingly, the correlation between vitamin B2 and acetate is inverse in the FVS and PLA groups; a positive correlation was found in the PLA group (*p* = 0.009) and a negative correlation in the FVS group (*p* = 0.038) (Figure 7B). The network-based analysis of the interaction microbiota, diet, and SCFA confirmed the positive correlation of 2-methyl butyrate with the genus *Faecalibacterium* (Figure 8).

## Discussion

The diet is well-known to modulate the gut microbiota composition and metabolism (33). In this study, we tested the antioxidant capacity, gut microbiota composition, and metabolic pathways in a group of healthy adult volunteers supplemented with a fruit and vegetable supplement for 6 weeks compared with a matched control group supplemented with a placebo. Typically, in the microbiome context, their health condition can be estimated by the gut microbial compositional homeostasis as the gut microbiome is proven to play a substantial role in the vicious cycle of diet-controlled pathophysiology, where the gut microbiome regulates physiology, and it is impacted by pathophysiology. Thus, any changes in the gut microbial composition clearly reflect the health or disease nature of humans. The measurement of gut microbial richness and abundance could be a vital indicator of human health. The literature review is strongly in favor of the diet as one of the factors that affect the gut microbial composition in terms of bacterial richness and abundance (34–37).

Interestingly, in this study, we observed that formulated fruit and vegetable supplementation contributes to modulate the gut microbial composition, compared with the placebo. The FVS showed a reduction in the relative abundance of genera from the Lachnospiraceae family, such as *Ruminococcus* and *Lachnobacterium*, and unclassified bacteria from the Erysipelotrichaceae family. Also, to a lesser extent, the genera *Faecalibacterium* and unclassified bacteria from the Lactobacillales order were affected (increased). A multiethnic study conducted by Frankenfeld et al. reported that the consumption of fruits and vegetables caused a gut microbial shift including the genera from Lachnospiraceae and Ruminococcaceae families (38). Results from different studies are controversial. The Medika study conducted by De Ioria et al. reported that the consumption of animal proteins showed a marked reduction in the relative abundance of *Lachnospiraceae* spp., and whether the intake of vegetable proteins, fiber, and potassium impacted the relative abundance of *Lachnospiraceae* spp. positively in the chronic kidney disease subjects (39). On the other hand, De Fillippis et al. demonstrated that the genus *Ruminococcus* from the Lachnospiraceae family (L-*Ruminococcus*) was positively affected by the animal-based diet and negatively by the vegetables–based diet (40). Thus, a complete vegetable and fruit-based diet or a formulated supplementation, like in our study, predominantly target the genera from the Lachnospiraceae family, especially *Ruminococcus* and *Lachnobacterium*.

The genus *Ruminococcus* is assigned to both Lachnospiraceae and Ruminococcaceae families, it is greatly affected by the dietary components (41), and it can play multiple biological and pathological roles, which are still not fully understood. For example, Zheng et al. conducted an interesting study in which they estimated the dietary inflammatory potential *via* the literature-derived index (DII) with the gut microbiota composition, and they reported that the species *R. torques* is associated with the pro-inflammatory diet group (42). Also, Smith-Brown et al. demonstrated that fruit intake, especially apple and pears, drastically reduced the relative abundance of *R. gnavus* (43). In addition, in one of our recent studies, we found that *Ruminococcus callidus* and other unclassified bacteria (Ruminococcaceae family) could protect from cardiovascular diseases in obese Qatari subjects, whereas species from the genus of L-*Ruminococcus* such as *Ruminococcus gnavus* and *Ruminococcus torques* did not involve in the protection from CVD (44). Furthermore, a study conducted by Singh et al. reported that vitamin D supplementation in the healthy Qatari subjects showed a greater increase in the abundance of *Ruminococcus bromii* (45). The different *Ruminococcus* species can probably explain the different behavior. Unfortunately, we were not able to discriminate the species in our sample. Therefore, further studies are needed to clarify the role of the *Ruminococcus* spp. in health and diseases.

In addition to this, we identified a decrease in an unclassified bacterium from the *Erysipelotrichaceae* family due to the FVS. The rationale behind this decrease might be the folic acid consumption as the blood folate level was significantly higher in the FVS-supplemented group than in the PLA group. In support of this notion, Cheng et al. demonstrated a high-methionine low-folate (HMLF) diet considerably increased the relative abundance of bacteria from the *Erysipelotrichaceae* family in diet-induced HHcy mice (46). In addition, folate has been demonstrated to alter the inflammatory responses *via* DNA methylation and DNA synthesis (47), and Moein et al. demonstrated that the serum folate level is associated with inflammatory markers and disease clinical activity, and its optimal presence would help reduce the inflammatory response in IBD patients (48). Of note, many studies reported that *Erysipelotrichaceae* family bacteria are abundant in colorectal and colon cancer, but the results are not consistent (49). Strikingly, *Erysipelotrichaceae* has been demonstrated to mediate the TNF-alpha-mediated Crohn’s disease (50). Moreover, the co-network analysis revealed that both L-*Ruminococcus* and unclassified bacteria from the *Erysipelotrichaceae* family positively regulate each other, with L-*Ruminococcus* having a dominant regulatory role in the *Erysipelotrichaceae* family bacteria. Thus, there is a strong possibility that the FVS could provide a long-term health benefit by managing the genus L-*Ruminococcus* abundance level.

The pro-inflammatory response is closely connected to oxidative stress as these two entities promote response of each other (51). Thus, in this study, the PLA-supplemented subjects were expected to have a low antioxidant capacity. It is a well-accepted fact that the consumption of fruits and vegetables increases the antioxidant capacity (52), and in this study, the antioxidant capacity measured by ORAC assay was higher (but not statistically significant) in the FVS-supplemented subjects than in the PLA group. Regarding the direct role of the gut microbiota in the antioxidant function, some *Lactobacillus* strains are known to exert beneficial effects due to their antioxidant activities. Probiotics, containing *Lactobacillus* strains, can produce various metabolites with antioxidant activity, such as glutathione (GSH), butyrate, and folate (53). Different mechanisms of antioxidant activity have been identified in the *Lactobacillus* strains: production of metabolites and antioxidant enzymes for reactive oxygen species (ROS) scavenging, upregulation of host antioxidant enzyme activities, downregulation of enzyme activities related to the production of ROS in the host, and regulation of pathways signaling related to host antioxidants and host intestinal flora (54–56). The ORAC assay is one of the best methods used to assess the antioxidant activities of different *Lactobacillus* strains (54). In our study, we observed an increase in plasmatic levels of folate, an increase of an unclassified genus from the order Lactobacillales and, consequently, we observed an increasing trend in the plasma antioxidant capacity of the subjects supplemented with the FVS.

The microbial metabolites, such as SCFAs, are demonstrated to be a key factor in the modulation of the inflammatory pathway, binding to specific receptors that regulate the immune function pathways (57–59). Among the measured SCFAs, we observed a nominal increase in the total SCFA levels, and a significant increase in 2-methylbutyrate positively correlated with the relative abundance of *Faecalibacterium*. 2-Methyl butyrate is a branched chain fatty acid derived from the intestinal metabolism of isoleucine, an essential branched amino acid (BCAA) (60). Gut microbiota is able to both produce and metabolize BCAA, and its levels are associated with insulin resistance (61), osteoporosis (62), and aging (63) *via* mechanisms involving the microbiota. A randomized controlled trial confirmed that the probiotic administration improves the serum amino acid concentration, including BCAA, in the subjects eating plant proteins (64). The microbiota shift that we observed in the FVS group can explain the increase in the 2-methylbutyrate level by enhancing the BCAA metabolism. Moreover, in our study, we observed an increase in the 2-methyl butyrate level and ORAC (increasing trend) in the subjects supplemented with the FVS compared with the placebo. We can speculate on a potential underlying mechanism that involves the gut microbiota and SCFA in regulating the antioxidant capacity activated by fruit and vegetable supplementation. Our hypothesis is supported by an observational study that explored the potential anti-inflammatory capacity of the SCFA in patients under hemodialysis, and it showed a negative association of 2-methyl butyrate with bone morphogenetic protein 6 (BMP-6), a biomarker of vascular calcification, and hypothesized the role of 2-methyl butyrate and other SCFAs in reducing cardiovascular risk in hemodialysis (65).

The pathway analysis showed an increase in the fructose and mannose metabolism and the pentose phosphate pathway in the subjects supplemented with the FVS compared with the placebo. Recent animal studies reported similar findings with increased fecal levels of mannose and decreased levels of fructose, among others, in rats supplemented with 1.5% of green tea polyphenols (66). The pentose phosphate pathway is also known to be affected by the microbial composition and prebiotics, as demonstrated in high-fat diet-fed mice treated with Huangjinya black tea, where a reduced lipid metabolism corresponded with many increased metabolic pathways, including the pentose phosphate pathway (67).

Our study has strengths and limitations. The strengths of this study are a randomized double-blinded study design, the supplement provided in stick packs to ensure an equal amount of nutrients to each subject, the complete set of microbial composition and metabolite analyses, the measurement of the ORAC, and finally, the exclusion of any diet effect. The main limitation of this study is the small sample size, which requires the confirmation of the findings from larger cohorts and the validation in inflammatory disease settings.

## Conclusion

**FIGURE 9:**
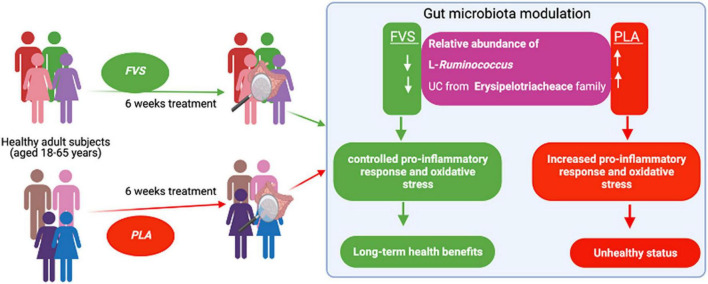
Schematic representation of the gut microbiota modulation by the FVS on healthy subjects.

In conclusion, we propose that the FVS can provide long-term health benefits by controlling the relative abundance of bacteria such as L-*Ruminococcus* and unclassified bacteria from the Erysipelotrichaceae family, possibly through the reduction of pro-inflammatory response (Figure 9). Further studies are required to confirm these findings and explore potential molecular mechanisms.

## Data availability statement

The data presented in this study are deposited in the NIH NCBI repository, accession number PRJNA833767.

## Ethics statement

The study was approved by the Ethical Committee Interaziendale Milan Area A and the approval number is 156/ST/2014. The patients/participants provided their written informed consent to participate in this study.

## Author contributions

LS and DC designed the study. ED, FP, and CB were involved in the subject recruitment process, diet data, and sample collection process. AM and ML performed blood tests. AT performed the data analysis for the diet and blood tests. AL processed stool samples and performed the gut microbiome data analysis. SM contributed to the gut microbiome data analysis. AT, AL, ML, and FP wrote the manuscript. All authors reviewed the manuscript.

## Footnotes

1
http://www.jerrydallal.com/random/randomize.htm

2
https://www.scottibassani.it/atlante-fotografico-delle-porzioni-degli-alimenti/
