# Supplementary material for: Modulation of gut microbiota: The effects of a fruits and vegetables supplement
Source: Front Nutr. 2022 Sep 23;9:930883. doi: 10.3389/fnut.2022.930883 (PMC9537686; doi:10.3389/fnut.2022.930883)
Supplement: Supplementary file 1 [file Data_Sheet_1.PDF]

Supplementary data – Figure 1S – study flow diagram

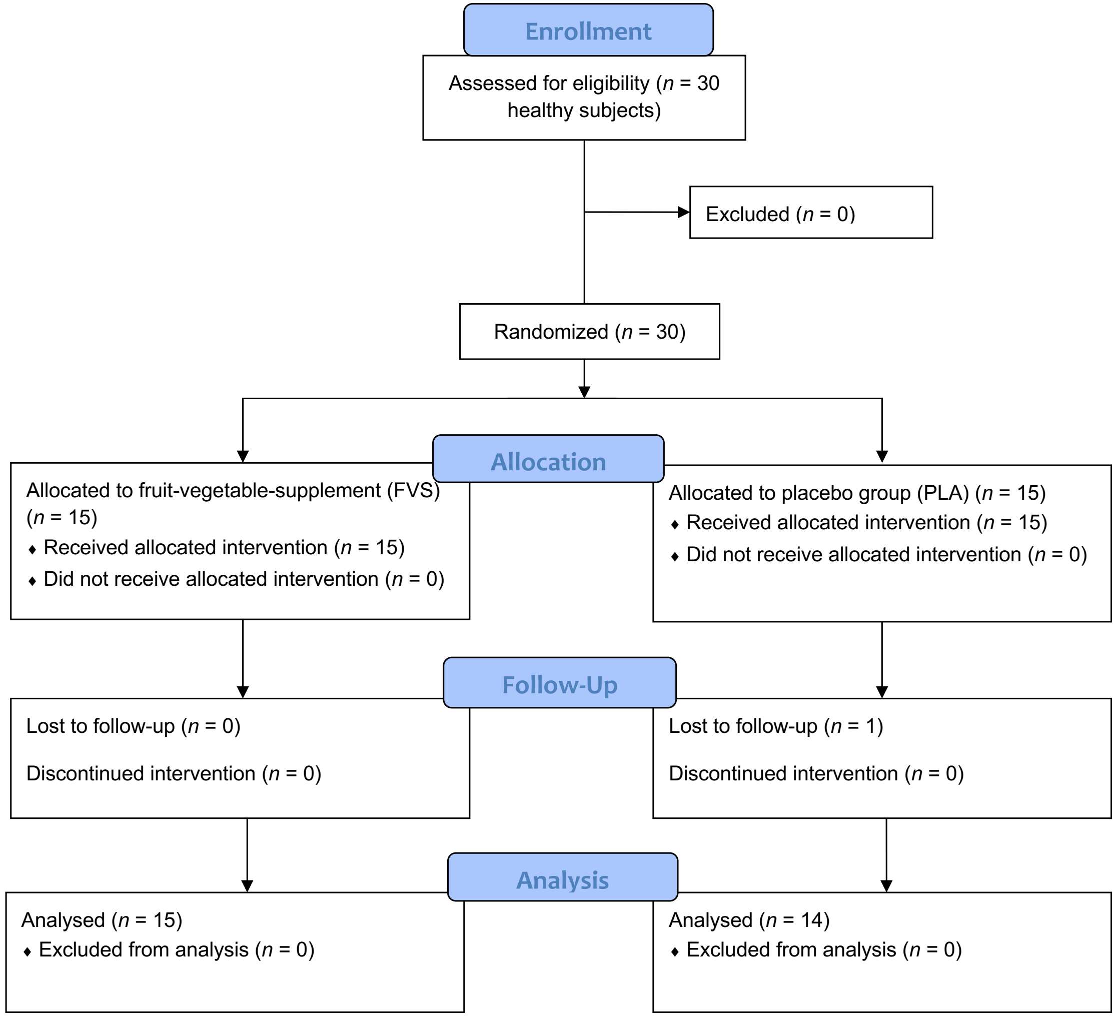

Supplementary data – Figure 2S - dietary intake

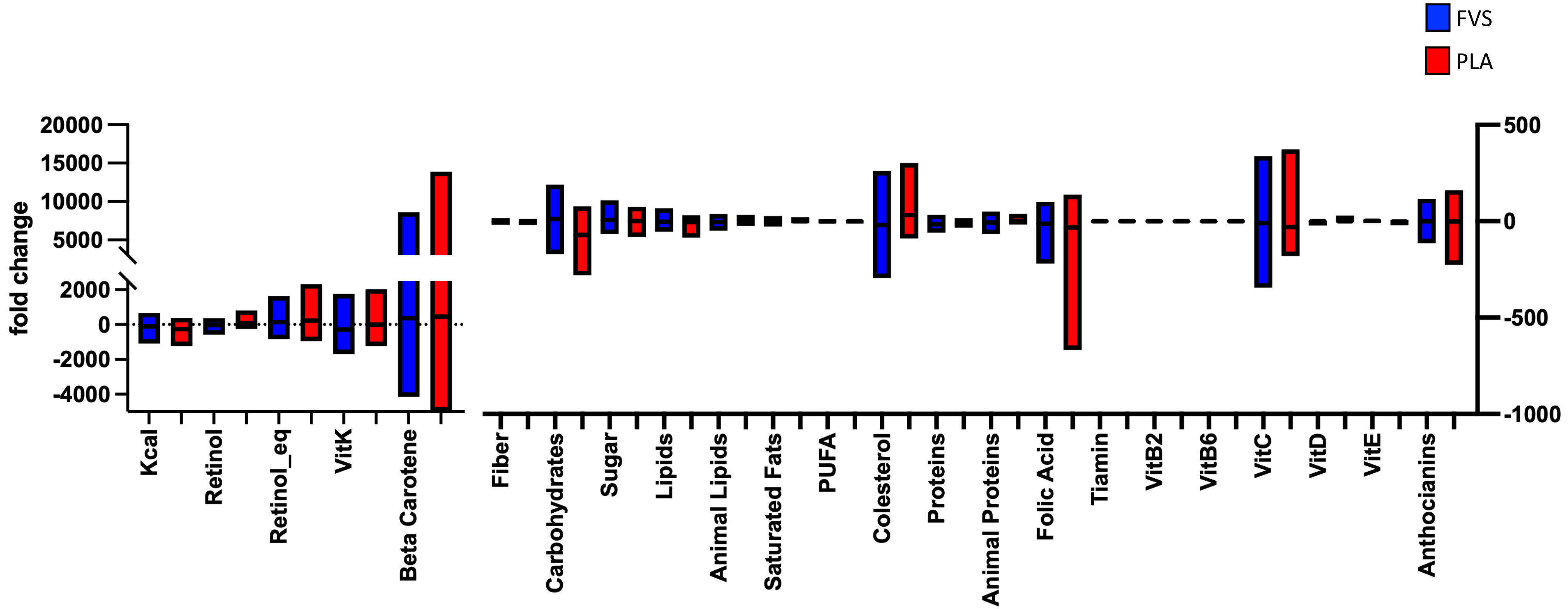

Supplementary data – Figure 3S – Rarefaction depth

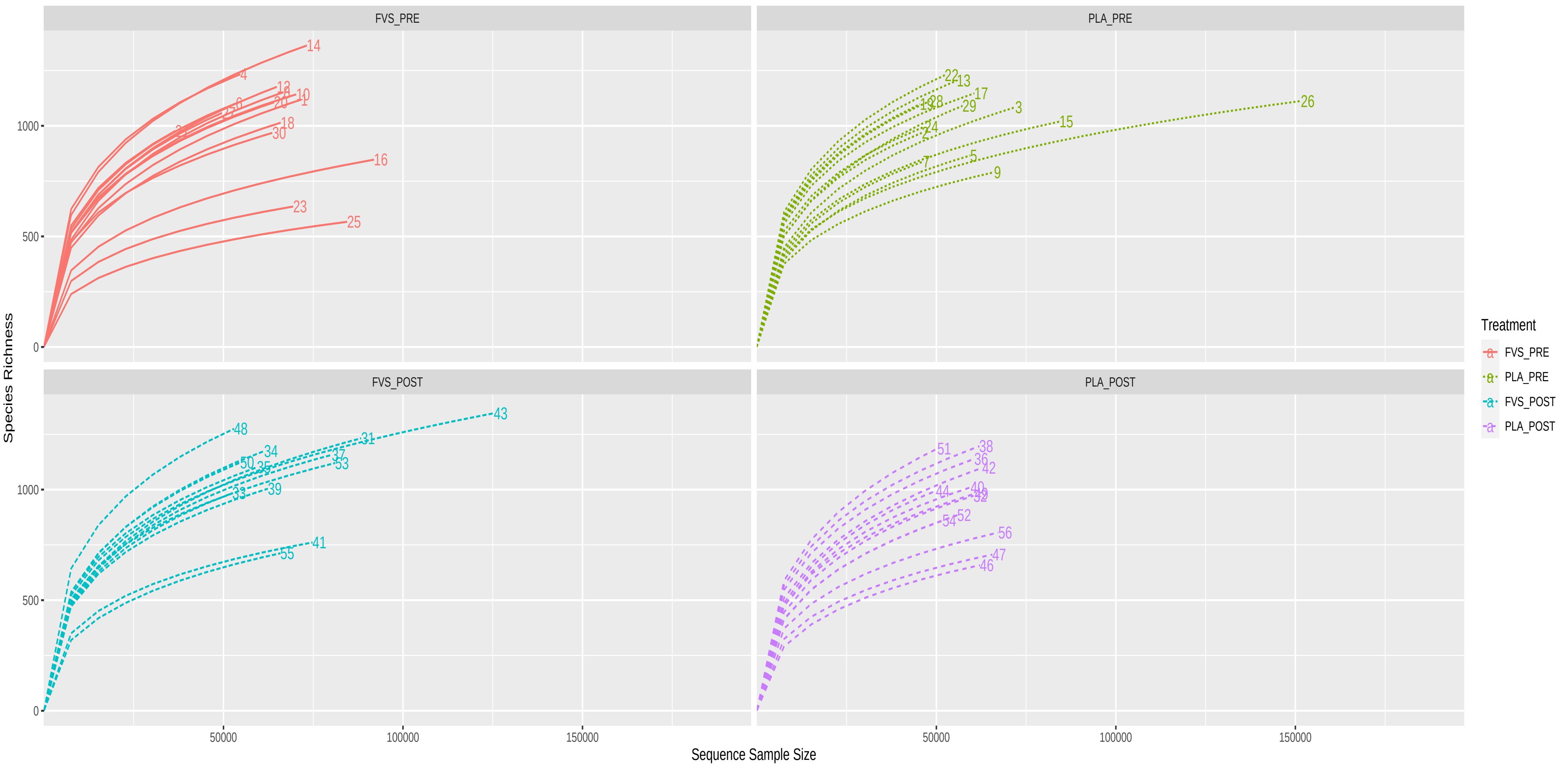

Supplementary data – Figure 4S – Analysis of composition of microbiomes (ANCOM) analysis and SIAMCAT analysis

(A) ANCOM analysis

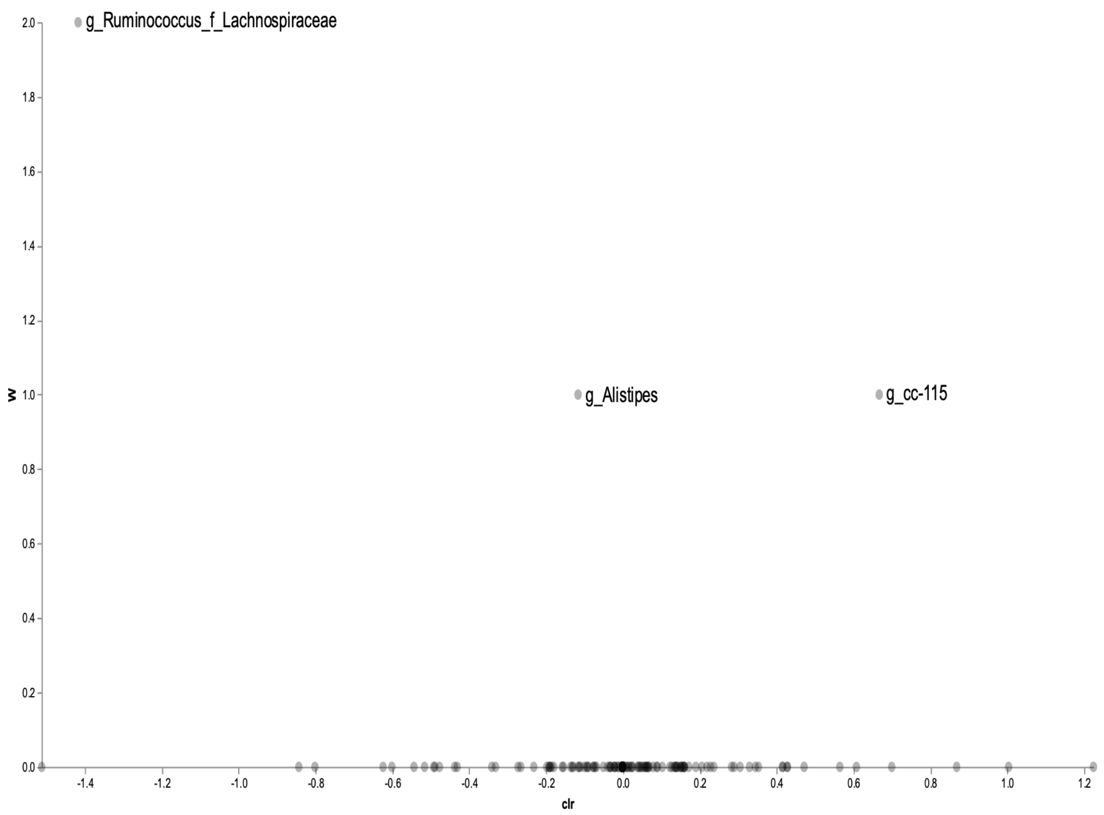

(B) SIAMCAT analysis

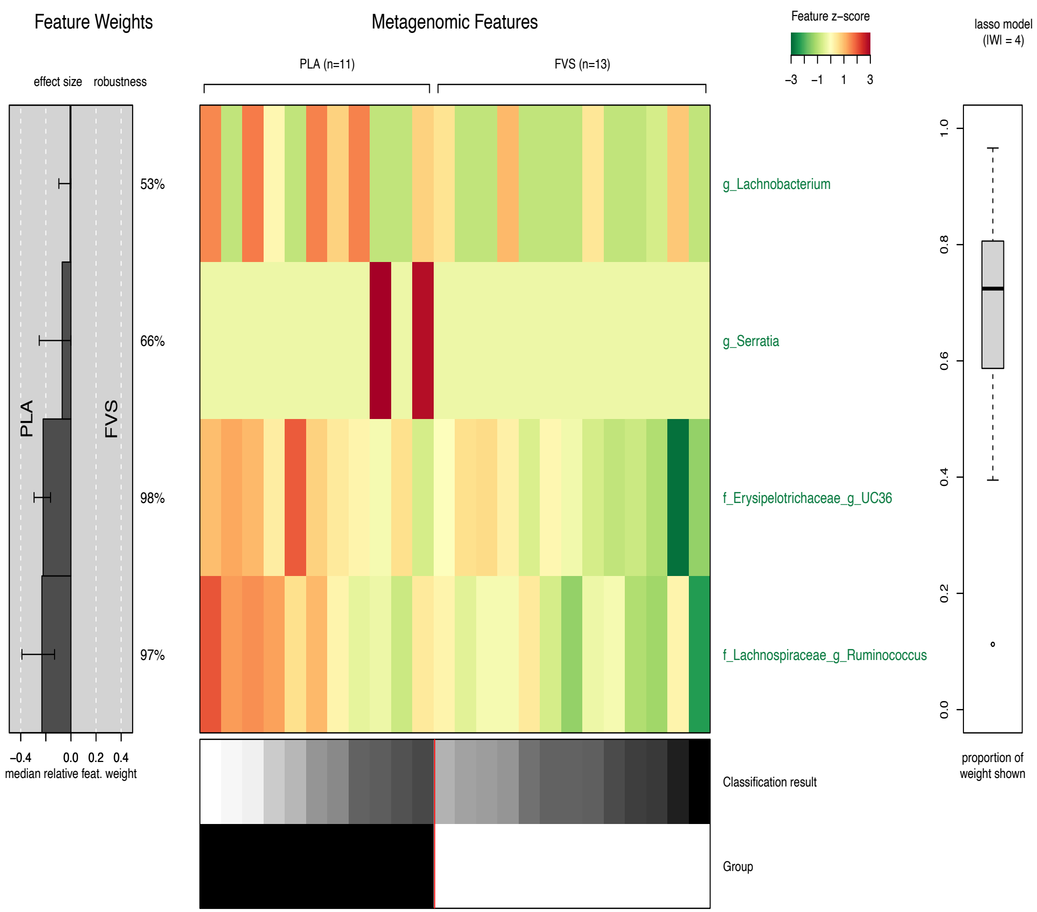

Supplementary data – Figure 5S - Microbiome profile

Phyla

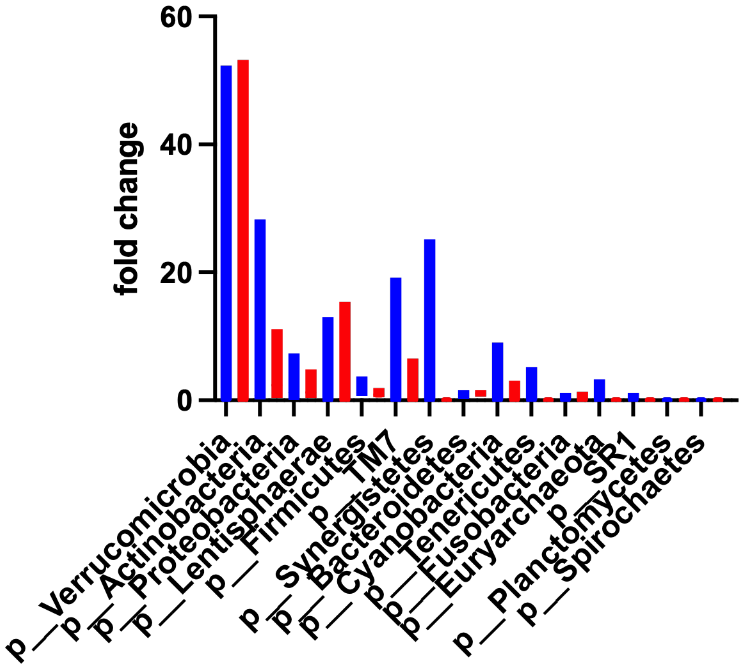

Top 30 genus

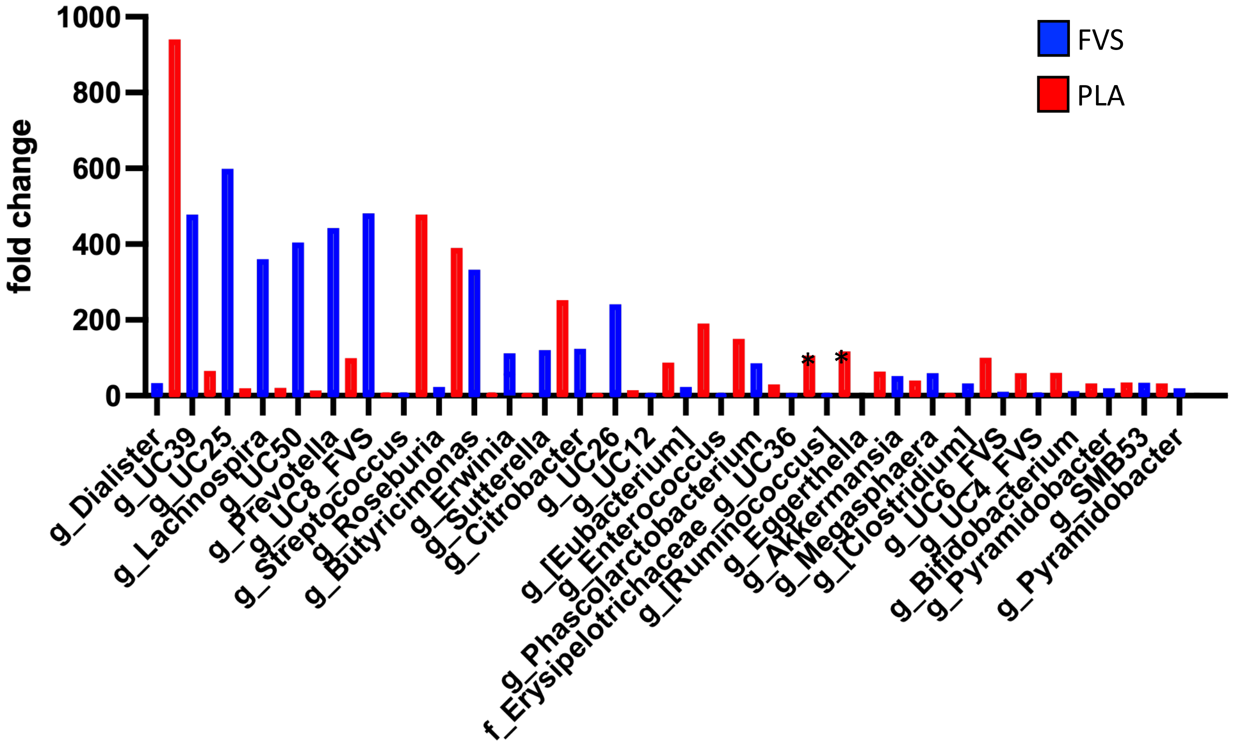

Supplementary data – Figure 6S - Microbiome profile. Significant bacterial fold changes in FVS and PLA groups

Data are shown as relative abundance (percentage of total gut microbiota composed of each phylum). The median and the interquartile (IQR) ranges are showed the boxplots. FVS, N=15, PLA, N=14. $^{*}p$<0.05, $^{**}p$<0.01, and $^{***}p$<0.001 when compared with the PLA group using Mann Whitney test.

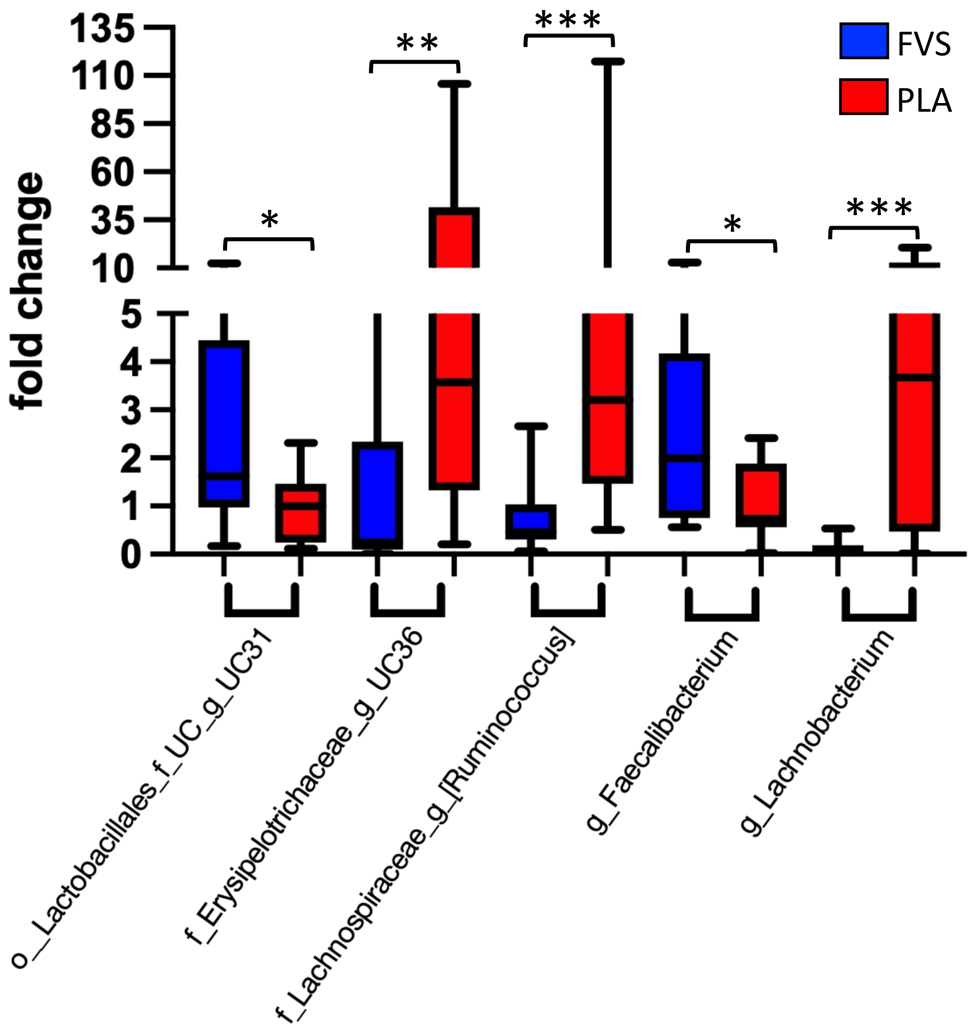

Supplementary data - Table 1S - FVS nutritional data and composition

Supplement Content	total/day (two sticks)	% RDA*
Carrot	1,16 g	
Fiber	800 mg	
Banana	400 mg	
Potassium	302 mg	15%
Broccoli extract<br>Containing sulforaphane	160 mg<br>480 mcg	
Apple	98 mg	
Kiwi	80 mg	
Blueberry extract<br>containing anthocyanin	60 mg<br>600 mcg	
Blood Orange extract<br>Containing anthocyanin	24,5 mg<br>686 mcg	
Tomato<br>Lycopene	20 mg<br>1,2 mg	
Vitamin E	12 mg	100%
Zinc	10 mg	100%
Vitamin B2	1,4 mg	100 %
Vitamin B1	1,1 mg	100 %
Beta carotene	490 mcg	
Folic Acid	261 mcg	130 %

*RDA: recommended daily allowance

CONCENTRATED FRUITS AND VEGETABLES FOOD SUPPLEMENT
(3 grams/stick)

Ingredients: Carrot powder (Daucus carota L. - root) (19.3%), potassium citrate, Dietary fiber (13.3%), Bulking agent: sorbitol, Herbs, acidifier: citric acid, banana powder (Musa sp. L. - fruit) (6.7%), thickener: xanthan gum, dry extracts of: Broccoli (Brassica oleracea L. var. gemmifera and happened - leaves) standardized to 0.3% in sulforaphane (2.7%), bilberry (Vaccinium myrtillus L. - fruit) standardized to 1% anthocyanins (1.0%), Blood Orange (Citrus sinensis L. var. dulcis - fruit) standardized to 2.7% anthocyanins (0.4%); apple powder (Malus domestica Borkh - fruit) (1.6 %), zinc gluconate, anti-caking agent: silicon dioxide, dust Kiwi (Actinidia chinensis L. - fruit) (0.8%), beta-carotene, vitamin E acetate (DL- alpha tocopheryl acetate , gelatin), Tomato powder (Solanum lycopersicum L. - fruit) standardized to 6% lycopene (0.3%), Sweetener: Sucralose and Acesulfame K, Riboflavin (Vitamin B2), Thiamine hydrochloride (Vitamin B1), Folic Acid. The extracts contain Maltodextrin.

The chemical composition of the placebo stick will be as follows:
Sorbitol, Flavor Citric Acid, Xanthan Gum, micronized silica, coloring: E110, Sucralose, Acesulfame K.
